# The relevance of psychosocial risk factors at work in the NR-1 Risk Management Program

**DOI:** 10.47626/1679-4435-2024-224

**Published:** 2025-01-13

**Authors:** Sergio Roberto de Lucca, Andrea Franco Amoras Magalhães

**Affiliations:** Editor-in-Chief of the Brazilian Journal of Occupational Medicine; Editor-in-Chief of the Brazilian Journal of Occupational Medicine

In the final issue of 2023, we announced that the *Revista Brasileira de Medicina do Trabalho* (RBMT) was joining SciELO and that we would be improving the editorial processes and editorial board to increase our journal’s impact and the quality of its articles on a national and international level.

With this fourth issue, we complete the 2024 collection and reinforce our commitment to increasing the diversity of articles published in the RBMT, which can serve as a reference and contribute to the debate on contemporary topics of interest to occupational physicians and other occupational health professionals. Therefore, this issue’s Editorial addresses the relevance of identifying psychosocial factors at work and the mandatory management of these factors by organizations as of May 2025.

Due to the importance of this debate, we announce a call for articles for a dossier on the relevance and experiences of managing psychosocial factors in public and private organizations, which will be published in the first issue of 2025.

Rapid changes in the world of work have changed job opportunities and work itself. In an increasingly competitive environment, new forms of management, individual performance demands and increasingly challenging goals have been affecting the physical and mental health of workers.

In the pursuit of short-term results, managerialism has increased tensions and atomized work relationships, resulting in dissatisfaction and suffering at work. This contributes to the rapid growth of mental and behavioral disorders.

Scholars and international organizations have associated the increase in these disorders with psychosocial risk factors at work, which are often poorly managed. In 1984, the World Health Organization and the International Labor Organization conceptualized psychosocial factors at work^1^ as the result of relationships and

“interactions between work, the work environment, job satisfaction, organizational conditions and the worker’s capabilities, needs, culture, personal situation outside of work, perceptions, and experiences that can influence health, performance, and job satisfaction.”

The Brazilian Ministry of Labor and Employment’s recent Ordinance 1419/2024^2^ included psychosocial risk factors at work as an integral part of the NR-1 Risk Management Program, assigning organizations the responsibility of assessing, implementing, and managing these factors. Previously, the Ministry of Labor and Employment had assigned the Internal Accident and Harassment Prevention Committee the obligation to participate in and monitor the management of moral and sexual harassment in the workplace.^3^

In the Ministry of Health’s updated List of Work-Related Diseases,^4^ psychosocial factors at work are considered the essence of work organization itself regarding “work management and content, work relations, work environment conditions, person-task interaction, violence, and moral/sexual harassment and discrimination.”

Although the NR-1 itself does not deal with psychosocial factors in detail, the diagnosis and management of these factors are essential to ensure a healthier work environment, requiring the participation of all levels of the organization, occupational physicians, and other occupational health professionals.

Psychosocial factors at work are generated by the organization of the work itself and include its content, workload and pace, hours and shifts, degree of autonomy, interpersonal relationships, support, leadership quality, communication and participation in decisions, conflicts at home and at work, and career development^5^ (Chart 1).

**Chart 1:** Psychosocial risk factors at work, described by the European Framework for Psychosocial Risk Management.

Work content	Lack of variety or short work cycles. Fragmented and meaningless work. Poor use of skills. High uncertainty. Continuous exposure to people while working.
Workload and pace	Work overload or underload. Machine-driven pace. High levels of time pressure. Continuous subjection to deadlines.
Work hours	Work shift. Night work. Inflexible work hours, unpredictable hours, long hours or hours with no social interaction.
Control	Low participation in decision-making. Lack of control over workload and work shift.
Environment and equipment	Problems with the reliability, availability, suitability, maintenance and repair of equipment and facilities. Poor environmental conditions, such as lack of space, poor lighting, and excessive noise.
Organizational culture and function	Poor communication. Low levels of support for problem-solving and personal development. Lack of definition and agreement on organizational objectives.
Interpersonal relationships at work	Social or physical isolation. Poor relationships with superiors and colleagues. Interpersonal conflicts. Lack of social support.
Role of the organization	Role ambiguity and conflict. Role insufficiency. Responsibility for people.
Career development	Career stagnation and uncertainty. Underor over-promotion. Low wages. Job insecurity. Low social value of work.
Work-home interface	Conflicting demands from work and home. Lack of support at home. Dual career problems.

Source: Leka & Cok, 2008^5^

Excessive workload, lack of control over tasks, frequent or poorly communicated changes, conflicts, moral or sexual harassment, and lack of social support among colleagues and supervisors can lead to occupational stress, anxiety, depression, and other psychological and musculoskeletal disorders, compromising health, quality of life, and work performance.

Therefore, it is essential to assess worker perception, both individually and collectively, regarding the main psychosocial factors that trigger stress and illness in organizations. This assessment should include a broad range of participants, involving all hierarchical levels of the organization and should maintain the participants’ confidentiality.

Several validated self-report instruments are available in Portuguese to collectively assess psychosocial factors.^6^ Complementing quantitative results with focus groups and individual interviews is also suggested.
